# Gut microbiota mediated hypoglycemic effect of *Astragalus membranaceus* polysaccharides in *db/db* mice

**DOI:** 10.3389/fphar.2022.1043527

**Published:** 2022-11-14

**Authors:** Qianbo Song, Sau Wan Cheng, Dan Li, Huiyuan Cheng, Yuen Sze Lai, Quanbin Han, Hoi Yan Wu, Pang Chui Shaw, Zhong Zuo

**Affiliations:** ^1^ School of Pharmacy, The Chinese University of Hong Kong, Hong Kong, Hong Kong SAR, China; ^2^ Institute of Chinese Medicine, The Chinese University of Hong Kong, Hong Kong, Hong Kong SAR, China; ^3^ School of Chinese Medicine, Hong Kong Baptist University, Hong Kong, Hong Kong SAR, China; ^4^ School of Life Sciences, The Chinese University of Hong Kong, Hong Kong, Hong Kong SAR, China

**Keywords:** Astragalus membranaceus polysaccharides, diabetes, gut microbiota, hypoglycemic mechanism, short chain fatty acids

## Abstract

Gut microbiota has been reported to be closely associated with Type-II diabetes. Restoration of disordered gut microbiota ecosystem has been developed into a therapeutic strategy and gradually applied on Type-II diabetes treatment with both western drugs and herbal polysaccharides. Although *Astragalus membranaceus* polysaccharides (AMP) have also been used to treat Type-II diabetes, no study investigated correlations between gut microbiota regulation and its hypoglycemic effect. In the present study, the role of gut microbiota on the hypoglycemic effect of AMP in *db/db* mice was investigated for the first time. Sixteen days treatment of AMP at the dosage of 600 mg/kg in *db/db* mice not only alleviated its diabetic symptoms significantly but also restored its gut microbiota community with increased production of fecal short chain fatty acids (SCFA). Our further Pearson correlation analyses revealed that the relative abundance of two intestinal bacteria, *Akkermansia* and *Faecalibaculum*, were significantly positively correlated with the hypoglycemic effect of AMP as well as fecal SCFA production. It was also noted that treatment of AMP resulted in increased secretion of glucagon-like peptide-1 (GLP-1) in serum and enhanced intestinal integrity. Further mechanistic study revealed that the increased SCFA after AMP treatment could stimulate GLP-1 secretion and improve intestinal integrity via enhancing the expression of G protein-coupled receptors 41/43 and tight junction proteins (Occudin and ZO-1), respectively, leading to the alleviation of diabetic symptoms in *db/db* mice.

## 1 Introduction

Dysbiosis of gut microorganism species was found to be associated with metabolic diseases such as diabetes, hyperlipidemia and hypertension (Jose et al., 2012). In recent years, an increasing number of clinical evidence showed that the dysbiosis of gut microbiota is closely related with T2D occurrence (Gurung et al., 2020). It was found that the abundance of producing-butyrate bacteria was decreased while that of reducing-sulphate bacteria was increased in T2D patients in comparison with those from healthy subject (Wang et al., 2012). Another clinical research also reported to observe the compositional and functional alteration of gut microbiota with the increased abundance of *Streptococcus* and decreased abundance of *Clostridiales* in T2D patients (Fredrik et al., 2013). Notably, the restoration of disordered gut microbiota ecosystem has been developed into a therapeutic strategy for T2D treatment in clinical practice (Adak and Khan, 2019).

Polysaccharides, as the bioactive macromolecular constituents of many medicinal herbs, have been broadly used in the therapy of T2D (Zheng et al., 2019). A growing number of preclinical studies demonstrated that certain herbal polysaccharides exhibited the antidiabetic effect via regulating the disordered gut microbiota community (Hu et al., 2018; Wu et al., 2019; Ma et al., 2022). Ginseng polysaccharides was observed to decrease the fasting blood glucose significantly and restore the dysbiosis of gut microbiota system by decreasing the abundance of *Proteobacteria* (Li et al., 2018). Dendrobium polysaccharides were also reported to be able to restore the disordered gut microbiota community by increasing the abundance of bacteria that could produce branched amino acids (Chen et al., 2021).

Recently, *Astragalus membranaceus* (Chao et al., 2009; Ko et al., 2014) and its polysaccharides fraction (Zheng et al., 2020) have also been reported to possess various kinds of biological activities including hypoglycemic effect. Although a few studies reporting that multiple pharmacological pathways may be involved in the hypoglycemic effect of *Astragalus membranaceus* polysaccharides (AMP) such as regulating GLUT-4 signal pathway (Liu et al., 2010) and inhibiting the expression of protein tyrosine phosphatase 1B (Mao et al., 2009). To the best of our knowledge, no studies investigated the role of gut microbiota in the hypoglycemic effect of AMP. Since gut microbiota has been reported to be associated with the hypoglycemic effect of herbal polysaccharides, it was hypothesized that gut microbiota might play an important role on the hypoglycemic effect of AMP. The present study is proposed aiming to investigate the role of gut microbiota in the hypoglycemic effect of AMP as well as its underlying mechanisms.

## 2 Materials and Methods

### 2.1 Materials

#### 2.1.1 Herbal materials

The dry roots of *Astragalus membranaceus* originated from Gansu, P. R. China was purchased from Fuming Dispensary (Tai Wai, Hong Kong, P. R. China). The voucher specimens of the herb were authenticated by Dr. Lau Tai Wai and kept in the Li Dak Sum Yip Yio Chin R&D Centre for Chinese Medicine at The Chinese University of Hong Kong.

#### 2.1.2 Chemicals and reagents

Glucose, rhamnose, mannose, arabinose, galactose, xylose, glucuronic acid, galacturonic acid, 1-phenyl-3-methyl-5-pyrazolone were purchased from Chroma Biotech Co., Ltd. (Chengdu, China). Acetic acid, propanoic acid, butyric acid and DPP4 inhibitor were purchased from Sigma-Aldrich (Saint Louis, MO, United States). Trimethyl acetic acid was purchased from Thermofisher (Waltham, MA, United States). Dextran standards (MW: 1200 Da, 4300 Da, 37600 Da, 121500 Da, 618300 Da, 2556000 Da) were purchased from American Polymer Standards Co., Ltd. (Mentor, OH, United States). Metformin was purchased from Meilun Biotech Co., Ltd. (Dalian, China). The purity of all reagents was more than 99%. Ethanol, chloroform, and methanol were all in HPLC grade and obtained from Merck KGaA (Darmstadt, Germany). Distilled water was supplied by a Millipore water purification system (Milford, MA, United States).

### 2.2 Preparation of *astragalus membranaceus* polysaccharides

The dry roots of *Astragalus membranaceus* (1.2 kg) were cut into slices and soaked into 95% ethanol (3 L) for 3 days to remove the fat followed by air-drying and extracting with water (18 L) for 3 h under 100°C in round bottom flask connected with a reflux condenser. The extraction was repeated for three times and the resulted extraction mixture was combined and centrifuged at 4500 rpm for 20 min under room temperature. The obtained supernatant was concentrated to 2.5 L under reduced pressure followed by addition of 10 L ethanol. The mixture solution was stood still at 4°C overnight and centrifuged (4500 rpm, 20 min) to obtain the pellet, which was re-dissolved into water (3.5 L) and dialyzed in the dialysis bag (cutting-off molecular weight: 3500 Da) against running water for 2 days. The resulted solution was lyophilized to obtain *Astragalus membranaceus* polysaccharides (AMP) by a Freezone freeze dryer (Labconco, MO, United States)

### 2.3 Chemical characterization of *astragalus membranaceus* polysaccharides

#### 2.3.1 Sugar content and protein residue content in our prepared *astragalus membranaceus* polysaccharides

The total sugar content was measured by anthrone-sulfuric acid method with glucose was selected as standard to plot the calibration curve according to the previous report (Leyva et al., 2008). In addition, the total protein residue content of the prepared AMP was measured by Bradford method with bovine serum albumin as standard as described before (Fu et al., 2019).

#### 2.3.2 Monosaccharide composition analysis

The monosaccharide composition of AMP was analyzed after 1-phenyl-3-methyl-5-pyrazolone (PMP) derivatization by HPLC method with modifications (Kuang et al., 2020). Briefly, 2 mg AMP sample was hydrolyzed with 1 ml trifluoroacetic acid (TFA, 2 mol/L) in a sealed tube at 100°C for 6 h. The mixture solution was evaporated to dryness and the hydrolysate was re-dissolved in 2 ml of distilled water for subsequent derivatization reaction with PMP. Briefly, about 100 μL hydrolysate solution (1 mg/ml) or standard monosaccharide solution (1 mg/ml) was mixed with 100 μL NaOH solution (0.6 mol/L) and 200 μL PMP solution (0.5 mol/L in methanol), followed by reacting at 70°C for 2 h and subsequent neutralization *via* addition of 100 μL HCl solution (0.6 mol/L) and 1 ml distilled water. After extracting the resulting mixture with 1.5 ml chloroform for five times, the aqueous layers were combined and filtered through 0.22 μm filter prior to HPLC analyses. The chromatographic analysis was carried out with an Agilent 1260 HPLC system (Santa Clara, United States) equipped with a diode array detector set at wavelength of 250 nm. The chromatographic separation was achieved with a BPS HYPERSIL C_18_ column (5 μm, 250 × 4.6 mm, Thermofisher) and a mobile phase consisting of acetonitrile and 0.1 mol/L phosphate buffer (pH = 6.85) (15.5:84.5, v/v).

#### 2.3.3 Molecular weight determination

The AMP sample or dextran standards with different molecular weight (MW: 1200 Da, 4300 Da, 37600 Da, 121500 Da, 618300 Da and 2556000 Da) was dissolved in the deionized water to prepare an aqueous solution of 10 mg/ml followed by filtering through 0.45 μm filter. The filtrate was injected into a thermofisher U-3000 HPLC system equipped with a charged aerosol detector (Thermofisher, MA, United States). The molecular weight distribution was analyzed with a TSK GMPWXL gel permeation column (7.8 × 300 mm, Tosoh Bio-science, Tokyo, Japan) and a mobile phase of 20 mM ammonium acetate at the flow rate of 0.6 ml/min as described previously (Xu et al., 2014).

#### 2.3.4 Fourier transform infrared spectrum analysis

About 2 mg of AMP sample was mixed with potassium bromide (150 mg) completely in the dry environment followed by detection with a Fourier transform infrared (FT-IR) spectrometer at a scanning range of 4000–550 cm^−1^ according to the previous method (Kuang et al., 2020).

### 2.4 Animals grouping and treatment to evaluate the hypoglycemic effect of *astragalus membranaceus* polysaccharides in *db/db* mice

Male *db/db* mice (BKS.Cg-Dock7m+/+Lepdb/J, *n* = 24, 6-week-old) and wild type C57BL/6J mice (6-week-old, *n* = 8) were purchased from Laboratory Animal Services Center of The Chinese University of Hong Kong. Animals were housed in the cages with free access to food and distilled water under the controlled environment (23°C–27°C; 45–55% humidity; 12 h light-dark cycle). The animal experimental protocol was approved by Animal Ethics Committee of The Chinese University of Hong Kong (Ref. No: 22/008/MIS).

After an acclimatization for 1 week, animals were randomly divided into four groups (*n* = 8 for each group) including wild type normal group (WT), diabetic control group (*db/db*), metformin group (MET) and *Astragalus membranaceus* polysaccharides group (AMP). Mice from WT group and *db/db* group were treated with 0.2 ml distilled water. Mice from MET group and AMP group received 250 mg/kg metformin and 600 mg/kg AMP, respectively. All the mice were treated by oral gavage once daily for consecutive 16 days with daily body weight monitoring.

On Day 13, after 4 h fasting, the insulin tolerance test (iTT) was conducted according to previously reported method (Ángela and Herminia, 2015). All mice were intraperitoneally injected with human insulin at the dose of 0.75 IU/kg and the blood drop was collected from tail vein at different timepoints (0, 30, 60 and 90 min) for measuring blood glucose level by a glucometer (Bayer, Germany). After an overnight fasting on Day 14, the blood was collected via tail vein into a tube without anticoagulants on Day 15 for fasting serum insulin level measurement by a commercial mouse insulin kit (Millipore, Darmstadt, Germany). Subsequently, intraperitoneal glucose tolerance test (ipGTT) was conducted by injecting glucose (0.3 g/kg) with analyzing the blood glucose level before and 30, 60, 90, 120 min post injection by a glucometer (Bayer, Germany) as reported before (Ángela and Herminia, 2015). On Day 16, fresh feces from all the mice were collected into sterile tubes before dosing and stored at -80°C till analysis. On Day 17, after fasting for 4 h, all the animals were orally administered with glucose (1.5 g/kg) and anesthetized at 30 min post administration, followed by withdrawing the whole blood into a tube (including 5 mM DPP4 inhibitor but without anticoagulants) for GLP-1 and cytokine measurement and being sacrificed to harvest the small intestine and colon to store at -80°C till further analysis.

### 2.5 Mechanistic studies on gut microbiota mediated hypoglycemic effect of *astragalus membranaceus* polysaccharides

#### 2.5.1 Measurement of serum levels of GLP-1 and cytokines

The levels of TNF-α, IL-6, IL-1β, IFN-γ, and GLP-1 in the serum were measured with the corresponding commercial ELISA kits (Invitrogen, MA, United States) according to the manufacturer’s protocol.

#### 2.5.2 Western blot analysis of GPCR41/43 and tight junction proteins

The collected small intestine and colon tissues (about 0.1 g) were homogenized in RIPA lysis buffer solution (Bio-Rad Lab, Hercules, CA, United States). After centrifugation for 20 min at 15000 rpm, the obtained supernatant was mixed with Laemmli sample loading buffer, followed by loading on 7.5–12% SDS-PAGE gels for electrophoresis. Then the proteins in the gel were transferred into PVDF membrane (Bio-Rad Lab, Hercules, CA, United States). After blocking with 5% bovine serum albumin, the PVDF membranes were incubated with anti-Occludin antibody (1:1000, Thermofisher, Cat. No: 711500), anti-ZO-1 antibody (1:500, Thermofisher, Cat. No: PA585256), anti-GPCR41 antibody (1:500, Thermofisher, Cat. No: PA575521), anti-GPCR43 antibody (1:300, Thermofisher, Cat. No: PA5111780), with the GAPDH as the internal control (1:10000, Abcam, Cat. No: ab181602) overnight at 4°C. The protein bands were detected by incubating with HRP-conjugated secondary antibody (1:1000, Abcam, Cat. No: ab97051) at room temperature for 1 h and quantified by ImageJ software.

#### 2.5.3 Short chain fatty acids content analysis by GC-MS

The collected fecal sample (100 mg) was homogenized in methanol (1 ml) by vigorous vortex for 3 min and sonicated for 10 min. The mixture was then centrifuged at 4°C (14000 rpm, 5 min) to obtain the supernatant, which was further diluted by 5-folds with methanol. The diluted supernatant (0.5 ml) was mixed with trimethyl acetic acid (internal standard, 5 μg/ml) solution (0.5 ml) and followed by injecting the mixture (1 μL) into Shimadzu QP2010 GC-MS system (Tokyo, Japan) for analysis. The analysis was carried out on an Agilent J & W fused silica capillary column (0.25 μm, 30 m) and the instrument parameters were set according to the reported method with modifications (Tsang et al., 2018). Briefly, the transfer line temperature and ion source temperature were 230°C and 200°C, respectively. High purity helium served as the carrier gas. The ionization mode was electron impact, with the electron energy of -70 eV and scanning time of 0.5 s. Mass fragments (m/z) in the range of 10–650 were monitored and identified in NIST Mass Spectral Library.

#### 2.5.4 Bacterial genomic DNA extraction and sequencing analyses by 16S ribosomal RNA

Bacterial genomic DNA was extracted from the collected fecal samples (180–220 g feces/each sample, stored at -80°C) by using the QIAamp^®^ PowerFecal^®^ Pro DNA Kit (QIAGEN, Hilden, Germany). 16S ribosomal RNA (rRNA) amplicon sequencing method was employed to amplify and analyze the gut microbiota composition in collected fecal samples. The V3-V4 regions were amplified by polymerase chain reaction (PCR) with the primers 341F (CCTAYGGGRBGCASCAG) and 806R (GGACTACNNGGGTATCTAAT) as we described before (Lyu et al., 2021). Sequencing libraries were generated on an Illumina platform. Paired-end sequencing were merged and filtered on Fastp software to obtain the effective tags. The effective tags were analyzed and conducted denoise with DADA2 method in QIIME2 software. The sequences with less than 5 units were filtered out to obtain the final Amplicon Sequence Variables (ASV) and the ASV dataset was analyzed in QIIME2 software to obtain species annotation and calculate the bacteria diversity.

### 2.6 Data analyses

The insulin resistance was evaluated by the homeostatic model assessment of insulin resistance (HOMA-IR) index, which was calculated as follows:

$$HOMA-IR=\frac{Fasting\;blood\;glucose\;level\;\left(mmol/L\right)\times Fasting\;insulin\;level\;\left(mIU/L\right)}{22.5}$$

Statistical analyses were performed using GraphPad Prism 5.01 software (GraphPad Software, Inc., CA, United States). One-way analysis of variance (ANOVA) with Turkey’s test was employed for multiple group comparisons. In addition, Pearson correlation analyses between relative abundance of gut microbiota and hypoglycemic effect index (ipGTT test result, HOMA-IR index, and fasting glucose level) or total SCFA in mice from WT, *db/db* and AMP groups were conducted with R software package (Version 4.1.1). Data was present as mean ± standard error of mean.

## 3 Results

### 3.1 Chemical characterization of *astragalus membranaceus* polysaccharides

The lyophilized AMP was a light-yellow powder with the extraction yield of 2.05% (w/w). The total sugar content and protein content of AMP were 71.82 ± 3.94% and 1.97 ± 0.43% (w/w, *n* = 3), respectively, indicating that carbohydrates were the main composition of the prepared AMP sample.

**FIGURE 1:**
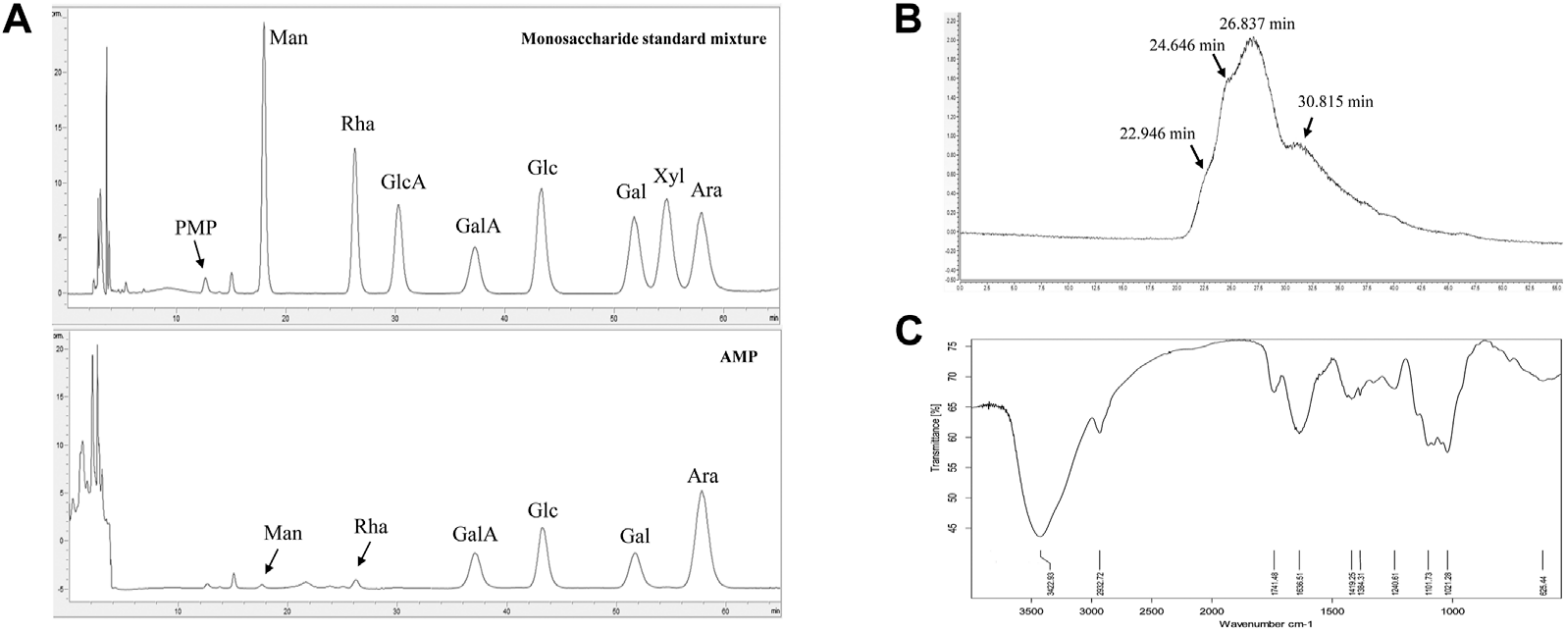
Characterizations of AMP with monosaccharide composition analysis **(A)**, molecular weight distribution **(B)** and FT-IR spectrometry **(C)**.

The monosaccharide composition analysis of our prepared AMP (Figure 1A) indicated that it was comprised of mannose, rhamnose, galacturonic acid, glucose, galactose, and arabinose, at their molar ratio of 0.12:0.16:1.00:1.06:0.73: 2.33. The HPGPC chromatogram of AMP (Figure 1B) showed four peaks, suggesting the average molecular weight of AMP were 3.7 kDa, 59.3 kDa, 274.5 kDa, and 900.7 kDa, respectively. FT-IR spectrum of AMP shown in Figure 1C suggested the following signal assignment: the signal at 3422.93 cm^−1^ was assigned to the stretching vibration bond of γ-hydroxyl group (Haxaire et al., 2003); the signal at 2932.72 cm^−1^ was assigned to the stretching vibration bond of *γ*-methylene group (Haxaire et al., 2003); the signal at 1741.48 cm^−1^ indicated the presence of carbonyl group (Zhou et al., 2018); the signal at 1636.51 cm^−1^ was the characteristic peak of water; the signal at 1419.25 cm^−1^ and 1384.31 cm^−1^ were assigned to the bending vibration bond of δ-methylene group (Liu et al., 2019); the signal at 1240.61 cm^−1^ was the stretching vibration bond of *γ*-C-O-C (Haxaire et al., 2003); the multiple signals around 1101.73 cm^−1^ indicated the presence of C-O (Haxaire et al., 2003) and the signal at 1021.28 cm^−1^ was the characteristic peak of pyranose ring (Liu et al., 2019).

### 3.2 Hypoglycemic effect of *astragalus membranaceus* polysaccharides on *db/db* mice

**FIGURE 2:**
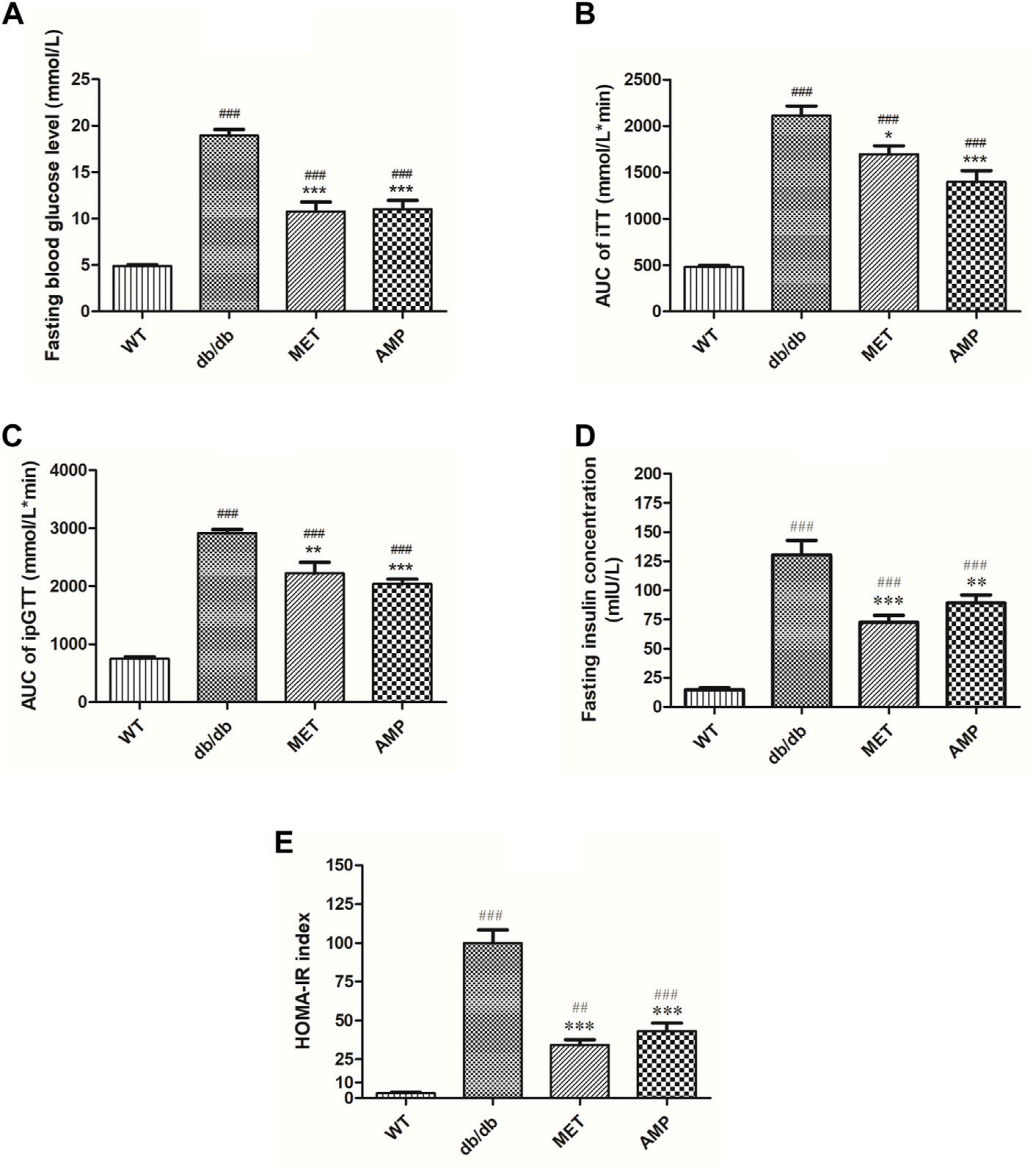
Evaluation of the hypoglycemic effect of AMP *via* monitoring of Fasting blood glucose level **(A)**, iTT test **(B)**, ipGTT test **(C)**, fasting insulin level **(D)**, and HOMA-IR index **(E)** in diabetic mice under different treatment (*n* = 8); #: *p* <0.05, ##: *p* <0.01, ###: *p* < 0.001 compared with WT group; *: *p* <0.05, **: *p* <0.01, ***: *p* <0.001 compared with *db/db* group.

As shown in Figure 2A, compared with the WT group (4.9 ± 0.1 mmol/L), mice from *db/db* group exhibited significantly higher fasting blood glucose level (18.9 ± 0.7 mmol/L), which indicated the obvious diabetic symptom. Compared among the *db/db* mice received different treatment, it was noticed that both AMP group (11.0 ± 0.9 mmol/L) and MET group (10.8 ± 1.0 mmol/L) exhibited significantly lower fasting blood glucose level. Similar trend was observed in the intraperitoneal glucose tolerance test as well (Figure 2B), *db/db* group (2915.1 ± 105.1 mmol/L*min) exhibited significantly higher blood glucose level compared with that of WT group (745.3 ± 16.9 mmol/L*min), after treatment with AMP or MET, the ipGTT result of *db/db* mice from AMP group (2039.0 ± 121.5 mmol/L*min) and MET group (2221.7 ± 88.0 mmol/L*min) were observed to decline significantly compared with that of *db/db* group. In the insulin tolerance test (Figure 2C), *db/db* group (2110.2 ± 105.1 mmol/L*min) exhibited significantly higher blood glucose level compared with that of WT group (480.0 ± 16.9 mmol/L*min). However, MET group (1698.8 ± 88.0 mmol/L*min) and AMP group (1396.7 ± 121.5 mmol/L*min) showed significantly lower level compared with that of *db/db* group. The fasting insulin measurement (Figure 2D) indicated that *db/db* group (130.40 ± 12.54 mIU/L) exhibited significantly higher insulin level compared with that of WT group (14.76 ± 1.74 mIU/L). After treatment with MET or AMP, the fasting insulin level of *db/db* mice from MET group (72.50 ± 6.04 mIU/L) and AMP group (89.18 ± 6.84 mIU/L) both significantly decreased compared with that from *db/db* group.

The calculated HOMA-IR index was the golden standard to evaluate the degree of insulin resistance. As shown in Figure 2E, it was observed that AMP group (43.4 ± 4.9) exhibited significantly lower HOMA-IR index compared with *db/db* group (99.03 ± 9.1), suggesting that AMP could significantly ameliorate the insulin resistance of *db/db* mice.

### 3.3 *Astragalus membranaceus* polysaccharides stimulated GLP-1 secretion and GPCR 41/43 expressions in *db/db* mice

**FIGURE 3:**
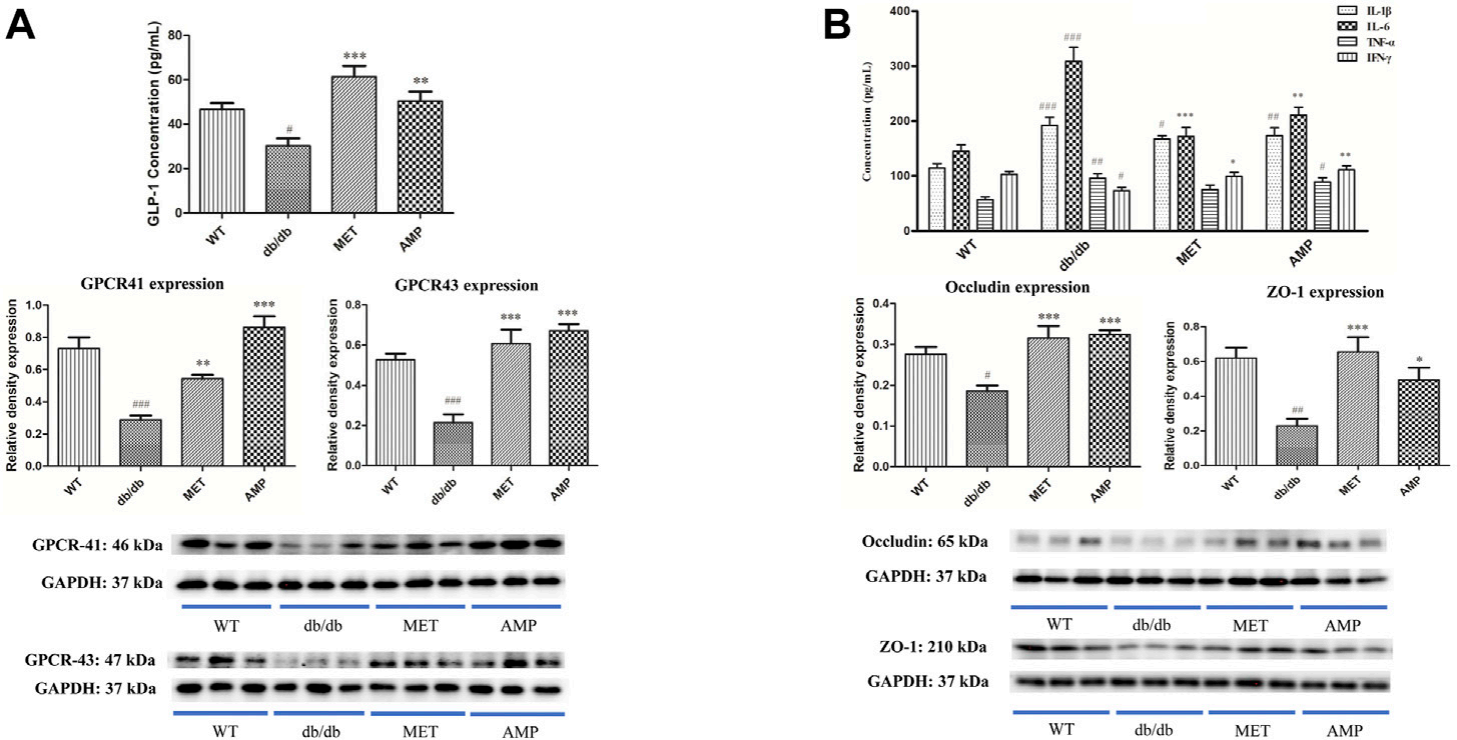
Increase of GLP-1 secretion and inhibition of serum inflammatory level by AMP *via* promoting the GPCR 41/43 expression in colon **(A)** and expressions of Occludin and ZO-1 in small intestine **(B)**, respectively. #: *p* <0.05, ##: *p* <0.01, ###: *p* <0.001 compared with WT group; *: *p* <0.05, **: *p* <0.01, ***: *p* <0.001 compared with *db/db* group.

As shown in Figure 3A, the Glucagon-like peptide-1 (GLP-1) level in the serum from WT group was significantly higher than that from *db/db* group. After treatment with AMP or MET, both AMP group and MET group exhibited significantly higher GLP-1 level compared with *db/db* group. In addition, G protein-coupled receptor 41/43 (GPCR 41/43), as the signal proteins which may be involved in the GLP-1 secretion, whose expression in the colon tissue was evaluated as well. As expected, the GPCR 41/43 expression in the WT group were significantly higher than that of *db/db* group. GPCR 41/43 in the AMP and MET group both exhibited significantly higher expression compared with *db/db* group. Notably, the GPCR 43 expression in AMP group was significantly higher than that of MET group, but no significant difference was observed on GPCR 41 expression between the two groups.

### 3.4 *Astragalus membranaceus* polysaccharides inhibited the serum IL-6 level and enhanced the expressions of ZO-1 and Occludin in *db/db* mice

As shown in Figure 3B, both AMP and MET can significantly decrease the IL-6 level compared with *db/db* group. Furthermore, AMP also could significantly increase the IFN-ϒ level compared with *db/db* group. Notably, both AMP group and MET group exhibited no significant difference on the IL-1β and TNF-α expression compared with *db/db* group. In addition, the expression of some tight junction proteins in the small intestine was evaluated as well. It was observed that *db/db* group exhibited significantly lower level on both ZO-1 and Occludin expression compared with WT group. However, after treatment with AMP or MET, both AMP group and MET group can significantly increase the ZO-1 and Occludin expression, which meant AMP and MET might improve the intestinal integrity and decrease the inflammation level of *db/db* mice.

### 3.5 *Astragalus membranaceus* polysaccharides increased the short chain fatty acids level in *db/db* mice

**FIGURE 4:**
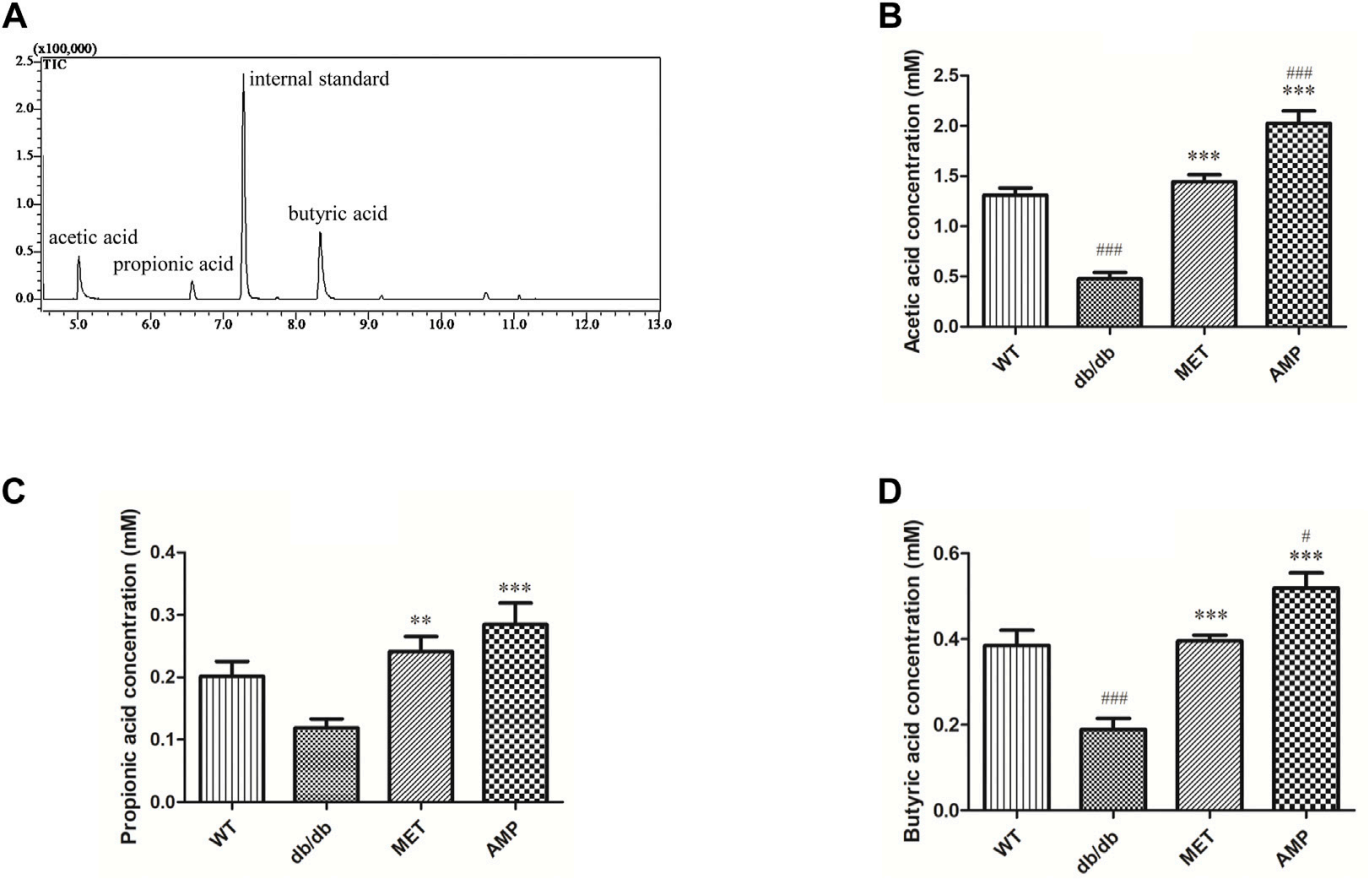
Comparison of fecal SCFA from *db/db* mice with different treatment (n = 8) as shown in the GC-MS chromatogram of SCFA extracted from feces **(A)**, acetic acid level **(B)**; propionic acid level **(C)** and butyric acid level **(D)**. #: *p* <0.05, ##: *p* <0.01, ###: *p* <0.001 compared with WT group; *: *p* <0.05, **: *p* <0.01, ***: *p* <0.001 compared with *db/db* group.

In the present study, trimethylacetic acid was selected as the internal standard for GC-MS analyses of SCFA due to its similar physiochemical properties and ideal retention time (Figure 4A). As shown in Figures 4B–D, it was observed that WT group exhibited significantly higher level on acetic acid and butyric acid, but with no significant difference on propanoic acid level, compared with *db/db* group. Furthermore, both AMP group and MET group exhibited significantly higher value on all the three SCFA level compared with the *db/db* group. Notably, AMP group exhibited significantly higher value (*p* <0.001) on acetic acid level compared with WT group or MET group. Besides acetic acid level, AMP group also exhibited significantly higher value (*p* <0.05) on butyric acid level compared with WT group or MET group. Collectively, AMP can significantly stimulate the generation of SFCA in the feces from *db/db* mice.

### 3.6 *Astragalus membranaceus* polysaccharides altered gut microbiota composition in *db/db* mice

**FIGURE 5:**
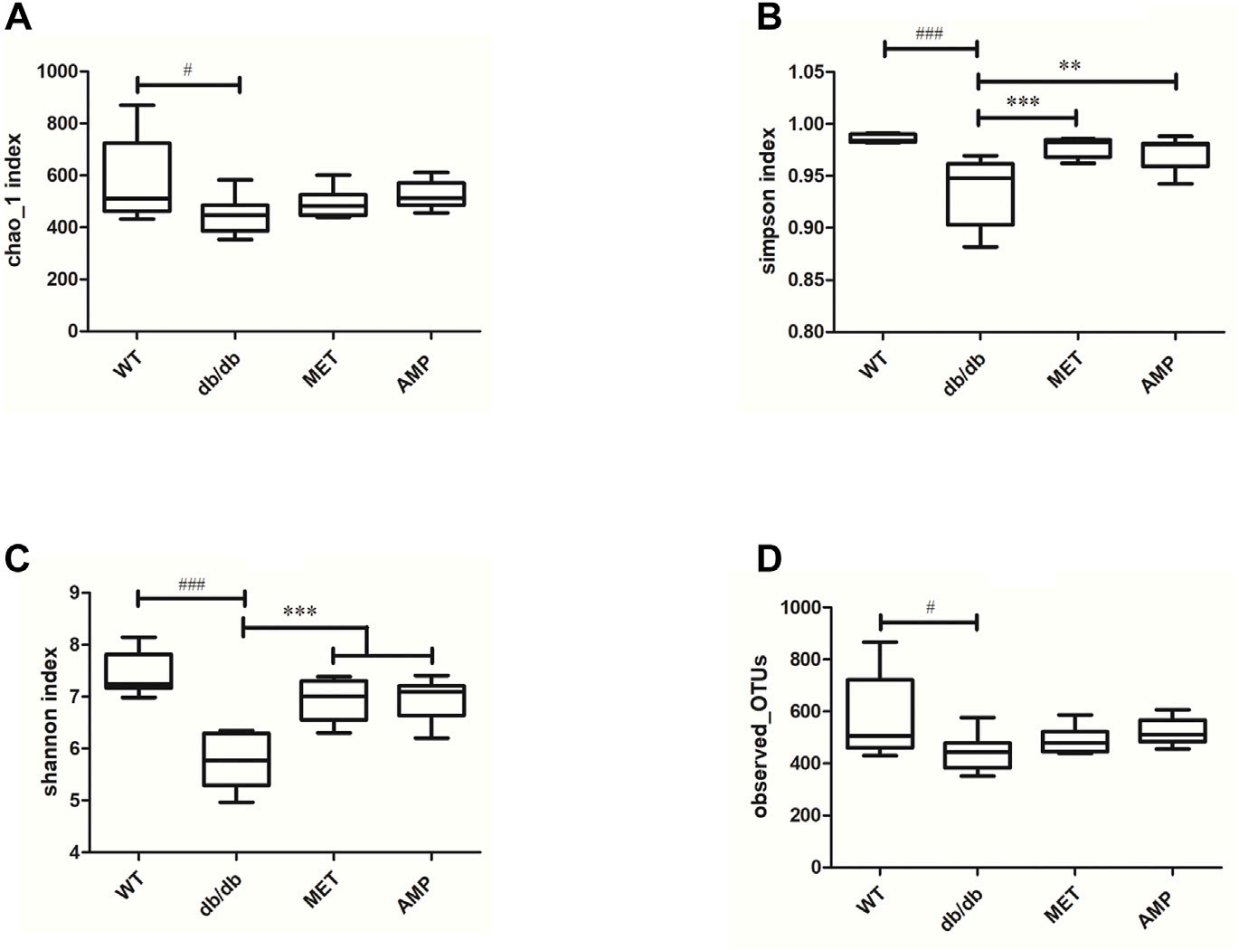
Comparison of biodiversity of gut microbiota community from C57BL/6J mice and *db/db* mice received different treatment (*n* = 8) *via* the Chao1 index **(A)**, Simpson index **(B)**, Shannon index **(C)** and Observed-OTUs **(D)**. #: *p* <0.05, ##: *p* <0.01, ###: *p* <0.001 compared with WT group; *: *p* <0.05, **: *p* <0.01, ***: *p* <0.001 compared with *db/db* group.

**FIGURE 6:**
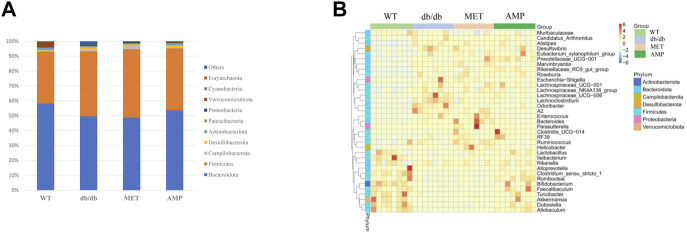
Comparison of relative abundance of gut microbiota community from C57BL/6J mice and *db/db* mice received different treatment as shown in relative bacterial abundance of top 10 at phylum level **(A)** and heatmap of top 35 at genus level **(B)**.

**FIGURE 7:**
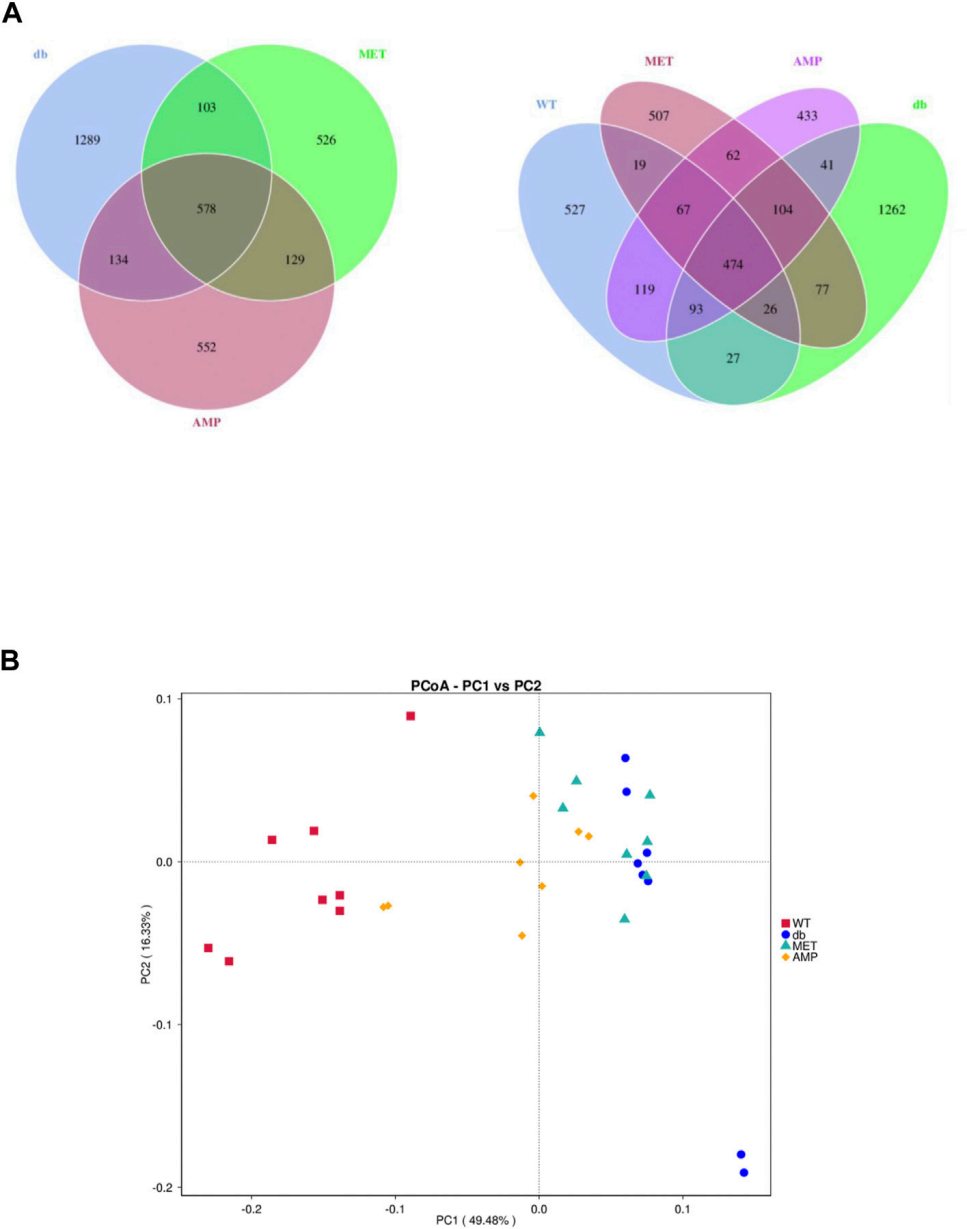
Difference in gut microbiota composition from C57BL/6J mice and *db/db* mice received different treatment (*n* = 8) as demonstrated in their Venn Diagrams of overlapping and exclusive ASVs clustering **(A)** and Principal Component Analysis **(B)**.

The richness and diversity of gut microbiota community in different groups were evaluated by biodiversity analysis as shown in Figures 5A–D *Db/db* group showed significant decline on the richness and diversity compared with WT group. After treatment with AMP or MET, Simpson index and Shannon index in both MET and AMP group were significantly higher than that of *db/db* group. Meanwhile, Chao1 index, Observed OTUs in MET and AMP group had no significant differences compared with *db/db* group, which meant MET and AMP could significantly improve the diversity of gut microbiota community in *db/db* mice but with no significant difference on the richness. Secondly, the relative abundance of intestinal bacteria at the phylum level (Figure 6A) was analyzed and it indicated that the *Bacteroidota* abundance in *db/db* group was lower than that in WT group. The heatmap of intestinal bacteria at the genus level (Figure 6B) also exhibited that the bacterial composition in each group were different. After treatment with AMP, the bacterial distribution tendency in *db/db* mice was reversed and became more like that of normal mice in some degree. In addition, the Venn diagram (Figure 7A) indicated that *db/db* group exhibited the greatest difference on ASV clustering compared with WT group, including 1484 exclusive ASV. However, AMP group showed the least difference on ASV clustering, with only 640 exclusive ASV, suggesting that AMP might decrease the difference of intestinal microorganisms between normal mice and diabetic mice. PCA analysis (Figure 7B) also exhibited that the points of *db/db* group were completely separated from those of WT group, whereas the points of AMP group were much closer with those of WT group, suggesting that the gut microbiota composition of AMP group was more similar to that of WT group.

### 3.7 Gut microbiota composition correlated with fecal short chain fatty acids production and hypoglycemic effect of *astragalus membranaceus* polysaccharides

**FIGURE 8:**
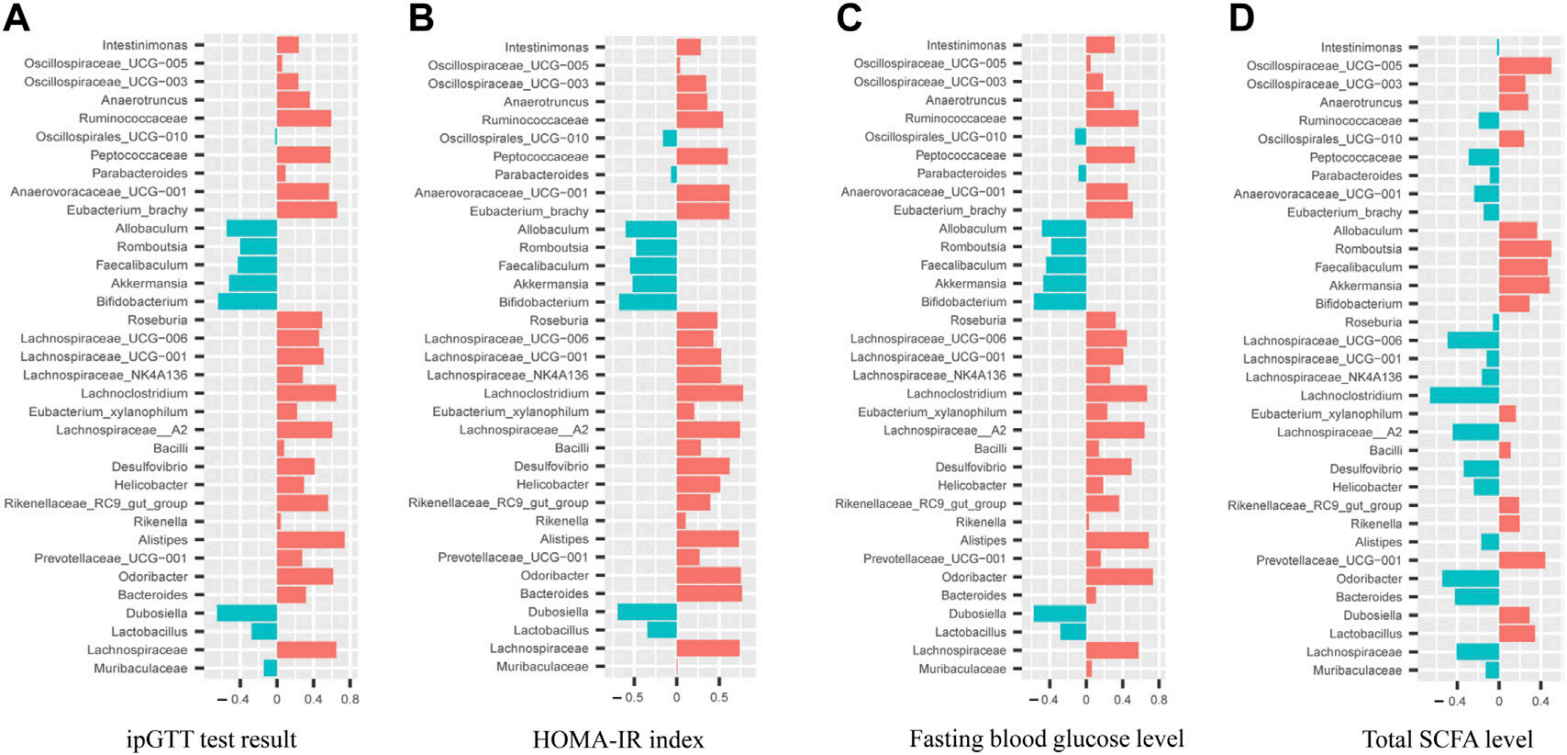
Correlation analyses between relative abundance of gut microbiota in mice from WT, *db/db* and AMP groups with their ipGTT test result **(A)**, HOMA-IR index **(B)**, Fasting blood glucose level **(C)**, Total SCFA level **(D)**.

Pearson correlation analyses between hypoglycemic effect index (fasting glucose level, ipGTT and HOMA-IR index) and the relative abundance of top 35 intestinal bacteria species were conducted as shown in Figures 8A–C. It was noted that the relative abundance of *Allobaculum*, *Faecalibaculum*, *Akkermansia*, *Bifidobacterium*, and *Dubosiella* were negatively correlated (*p<*0.05) with the hypoglycemic effect index with the correlation coefficients for *Bifidobacterium* and *Dubosiella* greater than 0.6. In addition, the relative abundance of 23 intestinal bacteria species exhibited positively correlation with the hypoglycemic effect index with correlation coefficients for *Odoribacter*, *Lachnospiraceae__A2*, and *Lachnoclostridium* greater than 0.6. Furthermore, the correlation analysis between total SCFA level and the relative abundance of top 35 intestinal bacteria species (Figure 8D) revealed that the level of total SCFA was positively correlated (*p<*0.05) with the relative abundance of *Akkermansia*, *Faecalibaculum*, *Romboutsia*, *Prevotellaceae_UCG−001*, and *Oscillospiraceae_UCG−005* with correlation coefficients all greater than 0.4. Meanwhile, the level of total SCFA was negatively correlated (*p<*0.05) with the relative abundance of *Odoribacter*, *Lachnoclostridium*, *Lachnospiraceae_UCG-006, Lachnospiraceae_A2*, and *Bacteroides* with correlation coefficients all greater than 0.4.

## 4 Discussions

Since increasing evidence demonstrated that the dysbiosis of gut microbiota was closely associated with T2D occurrence (Gentile and Weir, 2018), many therapeutic strategies designed based on alterations of gut microbiota have been gradually applied on the T2D treatment such as healthy feces transplantation, probiotics treatment and herbal polysaccharides treatment (Fan and Pedersen, 2021). Although AMP, as important chemical constituents of *Astragalus membranaceus*, have been reported to exhibit impressive potential on T2D therapy (Zou et al., 2009; Ye et al., 2014), our current study for the first time demonstrated its gut microbiota mediated hyperglycemic effect and disclosed the related underlying mechanisms. Our prepared AMP consisted of multiple monosaccharide compositions of galacturonic acid, glucose, galactose, and arabinose, which is similar to those reported in previous studies (Liu et al., 2019; Yang et al., 2021). The signal at 1741.48 cm^−1^ in the FT-IR spectrum also indicated the presence of carbonyl group, which was possibly due to the existence of galacturonic acid in our prepared AMP. It was noted that the molar ratio of galactose (13.5%) and arabinose (43.1%) in our prepared AMP was obviously higher than that in the previous study (Liu et al., 2019). Since our sample was extracted from planted herbs, but the reported sample was extracted from the herbal residue provided by commercial supplier. Therefore, it was speculated that the difference on monosaccharide composition might be resulted from different herb sources.

SCFA are the gut microbiota-producing metabolites derived from plant fibers or polysaccharides. Previous study reported that diabetic mice exhibited significant decline on SCFA level compared with normal mice (Han et al., 2021). In our current study, the level of all the three SCFA in the feces from *db/db* mice were increased significantly after treatment with AMP, suggesting that AMP could promote the production of SCFA, which were also observed in other antidiabetic herbal polysaccharides (Qi et al., 2022) including Dendrobium polysaccharides (Fang et al., 2022), Polygonatum polysaccharides (Zheng et al., 2020) and Ophiopogon polysaccharides (Wang et al., 2019). A growing number of evidence illustrated that SCFA were deeply involved in various physiological process like glycemic control and immunity modulation (Ley et al., 2005). GPCR 41 and GPCR 43, the two receptors mainly locating in colon, which can be activated selectively by SCFA (Brown et al., 2003). Although the function of GPCR 41/43 was still not illustrated very clearly, increasing studies indicated that they were the important signal proteins involving in glucose metabolism (Xiong et al., 2004; Bindels et al., 2013). GLP-1 is an intestinal hormone secreted by intestinal L cells and its physiological function is to stimulate insulin secretion and inhibit the appetite (Lewis et al., 2020). Previous studies reported that the increasing expression of GPCR41/43 proteins in colon could trigger the secretion of GLP-1. (Izumi et al., 2011; Gwen et al., 2012). Furthermore, an *in vitro* study demonstrated that SCFA might trigger the secretion of GLP-1 by enhancing the expression of GPCR 41/43 in the intestinal L cells (Kimura et al., 2013). In the present study, treatment of AMP in *db/db* mice not only lead to the increased level of three kinds of SCFA in fecal sample but also enhanced the GPCR 41/43 expression in the colon tissue as well as more GLP-1 was secreted, it indicated that the increased GLP-1 secretion stimulated by elevated GPCR 41/43 expression after AMP treatment might contribute to its hypoglycemic effect.

Some clinical studies found that the gut microbiota system of T2D patients contained higher concentration of detrimental intestinal metabolites such as indole, hydrogen sulfide, which may damage the intestinal integrity and result in the high level of inflammation factors *in vivo* (Zhao et al., 2018). Increasing evidence reported that some herbal polysaccharides were able to reshape the disordered gut microbiota community and maintain the intestinal health environment in diabetic animal model (Yan et al., 2017; Zhou et al., 2020). As the major metabolites derived from herbal polysaccharides, SCFA have been reported to exhibit impressive capability on improving intestinal microenvironment, enhancing intestinal integrity, and decreasing inflammation level (Greenblum et al., 2012). In this study, besides increased SCFA production level, treatment with AMP in *db/db* mice for the first time demonstrated to be able to improve the intestinal integrity by increasing the expression of tight junction proteins (Occludin and ZO-1) in small intestine and decrease the serum level of proinflammatory factor, like IL-6, TNF-α and IL-1β. Previous studies also found that increased intestinal integrity could improve the hyperglycemic symptoms by inhibiting the glucose and inflammatory factors influx in small intestine (Thaiss, C., et al., 2018; *Pearce* et al., 2013). Consequently, our study implied that the increased expressions of tight junction proteins (Occludin and ZO-1) might also contribute to the hypoglycemic effect of AMP.

To evaluate the impact of AMP on gut microbiota composition from *db/db* mice, a comprehensive analysis on gut microbiota composition as well as their function was conducted. It was found that the diversity of gut microbiota community was increased after treatment with AMP, but the richness exhibited no significant difference, which suggested gut microbiota composition became more diverse and complex under the treatment of AMP although the total number of bacteria did not increase. Diversity and richness were the two critical ecological index to evaluate the health of gut microbiota community (Cotillard et al., 2013). Previous research also found that the balanced and diverse gut microbiota system was beneficial in the prevention and therapy on chronic disease and metabolic dysfunction (Gentile and Weir, 2018), which was also supported by the current study. To investigate which intestinal bacteria was regulated by AMP, relative abundance analysis of gut microbiota was conducted. In the phylum level. It was observed that the ratio of *Bacteroidota*/*Firmicutes* in *db/db* mice (1.16 ± 0.07) was decreased compared with that in normal mice (1.76 ± 0.17). Meanwhile, the ratio of *Bacteroidota/Firmicutes* (1.52 ± 0.14) in *db/db* mice after treatment with AMP was increased. Although the ratio of *Bacteroidota/Firmicutes* was reported to correlate with the obesity and other metabolic disease, some contradictory conclusions also were observed when treated with different medicine (Patterson et al., 2016; Li et al., 2020). It was also evidenced by our findings that MET group (1.09 ± 0.10) and AMP group (1.52 ± 0.14) exhibited the contrary impact on the ratio, which implied that the regulatory effect of AMP and MET on the disordered bacteria community were different. The relative abundance analysis on the bacteria species in the genus level also demonstrated that the microbiota composition in different treatment groups was lack of similarity. Notably, the relative abundance of gut microbiota from mice in healthy state and diabetic state was obviously different, the similar phenomenon was also observed in T2D patients and healthy donors (Wang et al., 2012). The microbiota composition of AMP group was partially overlapped with that of WT group, which suggested AMP could partially restore the dysbiosis of gut microbiota community and reestablish the healthy intestinal microenvironment.

Our Pearson correlation analyses between hypoglycemic effect index and the relative abundance of gut microbiota revealed that *Allobaculum*, *Faecalibaculum*, *Akkermansia*, *Bifidobacterium*, and *Dubosiella* exhibited positively association with hypoglycemic effect significantly, while *Odoribacter*, *Lachnospiraceae__A2*, and *Lachnoclostridium* were found to be negatively correlated with the hypoglycemic effect significantly. Among these identified bacteria, *Bifidobacterium* is the most reported genus with the potential to protect gastrointestinal tract (Le et al., 2015) and improve glucose tolerance (Moya-Pérez et al., 2015; Aoki et al., 2017), *Faecalibaculum* were also found in lower abundance in T2D patients (Candela et al., 2016). *Akkermansia* were found to be beneficial in maintaining the intestinal integrity (Simpson et al., 2021), which was supported by our findings that treatment with AMP in *db/db* mice not only enriched the abundance of *Akkermansia*, also increased the expression of tight junction proteins. On the other hand, *Odoribacter* was also reported to exhibit higher abundance in diabetic mice and cause some health problems such as abdominal inflammation (Geurts et al., 2011). Our further correlation analyses between total SCFA level and the relative abundance of gut microbiota indicated that 18 intestinal bacteria including *Akkermansia*, *Faecalibaculum*, *Romboutsia*, *Prevotellaceae_UCG−001*, and *Oscillospiraceae_UCG−005* were positive correlated with SCFA level significantly. *Faecalibaculum* was found to be one kind of intestinal bacteria closely associated with the production of SCFA (Zagato et al., 2020). *Akkermansia* was the bacteria species with the function of utilizing non-digestible herbal polysaccharides and producing SCFA (Louis et al., 2014). In our study, treatment with AMP group in *db/db* mice also resulted in higher abundance of *Akkermansia*, *Faecalibaculum*, and *Romboutsia* significantly, which suggested that AMP might restore the disordered gut microbiota community by increasing the relative abundance of SCFA-producing bacteria. In summary, our current study served as the first attempt to investigate the role of gut microbiota on the hypoglycemic effect of AMP as evidenced by the positive correlation between the abundance of *Akkermansia*, *Faecalibaculum* and hypoglycemic effect as well as SCFA production, which provided an insight on the gut microbiota mediated hypoglycemic mechanism of AMP.

## 5 Conclusion

Our current study demonstrated that AMP exhibited the hypoglycemic effect and could restore the disordered gut microbiota community in *db/db* mice. Two intestinal bacteria, *Akkermansia* and *Faecalibaculum*, were disclosed to positively correlate with hypoglycemic effect and fecal SCFA production significantly. Mechanistic study found that the increased SCFA level in AMP treated *db/db* mice might stimulate GLP-1 secretion and improve intestinal integrity by enhancing expressions of GPCR41/43 and tight junction proteins (Occludin and ZO-1), respectively, which might be involved in the alleviating diabetic symptoms of *db/db* mice.

## Data Availability

The datasets presented in this study can be found in online repositories. The names of the repository/repositories and accession number(s) can be found below: https://www.ncbi.nlm.nih.gov/bioproject/PRJNA880037.
